# Management, Survival, and Costs of Pancreatic Cancer: Population-Based Observational Study in Catalonia

**DOI:** 10.3390/ijerph20095673

**Published:** 2023-04-28

**Authors:** Laura Guarga, Noelia Paco, Paula Manchon-Walsh, Emili Vela, Joaquim Delgadillo, Caridad Pontes, Josep Maria Borràs

**Affiliations:** 1 Catalan Health Service (CatSalut), 08007 Barcelona, Spain; laura.guarga@catsalut.cat (L.G.); npaco@catsalut.cat (N.P.); evela@catsalut.cat (E.V.); cpontes@catsalut.cat (C.P.); 2 Department of Pharmacology, Therapeutics, and Toxicology, Autonomous University of Barcelona, 08193 Barcelona, Spain; 3 Catalan Cancer Plan, Department of Health, Hospitalet del Llobregat, 08908 Barcelona, Spain; pmanchon@iconcologia.net; 4 Bellvitge Biomedical Research Institute (IDIBELL), 08006 Barcelona, Spain; 5 Digitalization for the Sustainability of the Healthcare System (DS3), Bellvitge Biomedical Research Institute (IDIBELL), 08006 Barcelona, Spain; 6 Blood and Tissue Bank, 08005 Barcelona, Spain; joaquin.delgadillo@gmail.com; 7 Clinical Sciences Department, University of Barcelona, Campus de Bellvitge, 08907 Barcelona, Spain

**Keywords:** pancreatic cancer, real-world data, early diagnosis, overall survival, treatment cost, oncogeriatric

## Abstract

Aim: Few published studies comprehensively describe the characteristics of patients with pancreatic cancer and their treatment in clinical practice. This study aimed to describe the current clinical practice for treating pancreatic cancer in Catalonia, along with the associated survival and treatment costs. Methods: A retrospective observational cohort study in patients diagnosed with pancreatic cancer from 2014 to 2018, using data from the healthcare records of the Public Health System of Catalonia, was conducted. Treatment patterns and costs were described by age groups from 2014 to 2018, with survival reported until December 2021. Results: The proportion of patients receiving surgery with curative intent was low, especially in older patients (23% of patients <60 years and 9% of patients ≥80 years). The percentage of patients treated with drugs for unresectable disease also decreased with age (45% of patients <60 years and 8% of patients ≥80 years). Although age was associated with significant differences in survival after curative surgery, no differences attributable to age were observed in patients who received pharmacological treatment for unresectable disease. In patients under 60 years of age, the mean cost of the first year of treatment was EUR 17,730 (standard deviation [SD] 5754) in those receiving surgery and EUR 5398 (SD 9581) in those on pharmacological treatment for unresectable disease. In patients over 80, the mean costs were EUR 15,339 (SD 2634) and EUR 1845 (SD 3413), respectively. Conclusions: Half of the patients diagnosed with pancreatic cancer did not receive specific treatment. Surgery with curative intent was associated with longer survival, but only 18% of (mostly younger) patients received this treatment. Chemotherapy was also used less frequently in patients of advanced age, though survival in treated patients was comparable across all age groups, so careful oncogeriatric assessment is advisable to ensure the most appropriate indication for eligibility in older patients. In general, earlier diagnosis and more effective pharmacological treatments are necessary to treat frail patients with high comorbidity, a common profile in older patients.

## 1. Introduction

Pancreatic cancer was the seventh most common cancer in Europe and the fourth leading cause of cancer-related death in 2020, with a standardized mortality rate of 18.6 per 100,000 population [1]. The high mortality could be explained because approximately 80% to 85% of patients were diagnosed at advanced stages, when surgical treatment is contraindicated [2]. Thus, the five-year survival might have been less than 7% in all patients in Europe during the period 2000–2007 [2,3], and might have reached 20% in cases where the tumor could be resected [4]. In Spain, there were an estimated 8218 new cases of pancreatic cancer in 2020, and this figure is expected to increase by 45% by 2040 [1].

Over 80% of pancreatic carcinomas could be caused by sporadic mutations, while 10% to 15% could be due to germline mutations [2,5,6,7,8]. In general, pancreatic ductal adenocarcinoma is the most frequent type (≥80%), followed by neuroendocrine tumors of the pancreas [2,3,8]. These tumors are often diagnosed in older people [2,3,9], with approximately 70% of new cases occurring in patients over 65 years of age in Europe [1]. Age is therefore an important risk factor for the development of this tumor, and it is also a consideration in decisions on the best therapeutic approach to follow [7,10,11,12]. However, chronological age alone is not the most appropriate measure to estimate life expectancy and/or functional reserve, so treatment decisions in geriatric cancer patients are often informed by a comprehensive geriatric assessment (CGA), which evaluates comorbidities, psychosocial disorders, nutritional status, quality of life, and concomitant treatments in addition to age [10,13,14].

As is mentioned in the Bratislava Statement, in recent years, no specific strategies have been developed for the primary prevention of pancreatic cancer [15], with the exception of smoking prevention measures, which have a limited impact on this tumor [16,17]. This situation could be associated with the lack of knowledge on specific modifiable risk factors or biomarkers that allow for early detection of the disease [5,15,18,19].

Surgery continues to be the most effective option when the tumor is resectable or becomes so after neoadjuvant treatment [2,18]. Some countries have centralized these procedures with the aim of improving the clinical outcomes in patients with pancreatic cancer, reorganizing health services, and changing professionals’ specializations [20].

For years, in Europe, pharmacological treatment has been based on chemotherapy schemes; these are selected according to the therapeutic objective (neoadjuvant or adjuvant use, increased survival and quality of life) and the patient’s baseline status. Reference regimens include combinations of 5-fluorouracil (5-FU), folinic acid, irinotecan, and oxaliplatin (FOLFIRINOX), gemcitabine plus paclitaxel, nab-paclitaxel, or capecitabine; gemcitabine monotherapy; and capecitabine plus radiotherapy [2,18]. In some palliative situations, palliative radiotherapy and surgical interventions could be used to treat local abdominal complications [2,18]. Over the last decade, no new drugs have been added to the recommendations in the European and Catalan Clinical Practice Guidelines, so for the vast majority of patients with advanced disease, chemotherapy remains the best available treatment [2,18].

However, treatment outcomes in these patients show low success rates. Surgery is effective only for early disease, and the available drugs provide little clinical benefit, probably because of the chemoresistance of this disease [21] and the toxicity of chemotherapy regimens. The risk/benefit ratio is therefore suboptimal, especially in older patients, who have worse tolerability due to greater frailty, more comorbidities, and age-related pharmacodynamic changes [10,14].

In addition, this neoplasm is a clear example of a neglected cancer, with little presence in health system planning, few efforts devoted to research for improving pancreatic cancer care [15], and a low representation of the oncogeriatric population in clinical trials [10,12]. The economic impact of pancreatic cancer treatment is mainly derived from the cost of hospitalization, followed by radiological, surgical, and chemotherapy interventions [22]. Some European countries, such as Sweden or Germany, estimate a cost for the entire treatment of EUR 16,066 to EUR 31,375 per patient [22].

Studies based on real-world and/or observational data are increasingly used for estimating the burden of morbidity in cancer patients, evaluating the efficacy of screening programs and new treatments, and for care planning at the national and regional levels [23]. This type of study makes it possible to describe and assess clinical practice to support decision-making around healthcare management [24,25,26]. Few published studies comprehensively describe the characteristics of patients with pancreatic cancer and their treatment in clinical practice [22]; the existing ones focus mainly on one drug or group of drugs or on some tumor subtype [21,27,28,29]. A comprehensive analysis of pancreatic cancer management according to patient age can help to characterize the effects of both pharmacological treatments and surgery on patient outcomes and the costs that they entail for the health system, helping to identify unmet needs and to support decision-making around the organization and financing of care.

The aim of this study was to describe the treatment patterns, survival, and treatment costs in patients diagnosed with pancreatic cancer from 2014 to 2018 in Catalonia.

## 2. Methods

This retrospective observational study was performed following the STROBE criteria [30]. Data on treatment patterns and costs were drawn from different healthcare registries in Catalonia for the period from January 2014 to December 2018, and survival was reported until December 2021.

### 2.1. Data Source

Spain has universal healthcare coverage, with medicines financed through the same reimbursement scheme across the country. That said, the management of the National Health System is fully devolved to the regions, which have heterogeneous arrangements for the organization and financing of their respective health systems [31]. The Catalan Health Service operates through a multiprovider system, known as the Integrated Public Health System of Catalonia (SISCAT), contracting healthcare services. It includes 68 hospitals, serving a population of 7.7 million people [32]. Electronic medical records with data on clinical practice are centrally managed, and these sources were used to draw data for this study [32,33,34]:

The hospital discharge minimum basic data set, which contains data related to acute hospital care and surgery;

Hospital outpatient drug registry, with clinical data on drug prescriptions from different therapeutic areas (excluding chemotherapy);

The Datamart Billing Service, which provides billing data for hospital drugs provided on an outpatient basis (including chemotherapy);

The Central Registry of Insured Persons, which is a repository of demographic data on all people covered by CatSalut.

The centralized registries lack some relevant information, for example, tumor-related variables (histology, stage, biomarkers), clinical characteristics (functional status, comorbidities), and prescribed treatments (indication for chemotherapy or palliative radiotherapy). Similarly, the registries do not contain information on palliative treatment delivered in long-term centers or outside acute hospitals, and there is not any centralized billing record detailing the cost of palliative care procedures or social and health care interventions in settings other than primary care or acute hospitals.

### 2.2. Study Population

Patients with a diagnosis of pancreatic cancer (International Classification of Diseases, 10th revision [ICD-10], codes C25; Supplementary material Table S1) on their hospital discharge report from 2014 to 2018 were eligible [35]. We selected this study period in order to enable sufficiently lengthy follow-up and to assess survival according to treatment patterns.

Information on treatment strategy was collected from the hospital discharge minimum basic data set, hospital outpatient drugs registry, and the Datamart Billing Service. Each case was assigned a unique anonymous identifier to combine the data for patients treated in different hospitals. The Agency for Health Quality and Assessment of Catalonia (AQuAS) provided the data in the framework of the PADRIS Program.

Approximately 10% of patients who underwent surgery received at least some of their treatment from private providers, although it is likely that some returned to the public system for systemic treatment. However, the analysis included only the data from the public health care system.

### 2.3. Outcomes

The sample was described according to the following variables: sex, age group (<60 years, 60–69 years, 70–79 years and ≥80 years), tumor site, and year of diagnosis. Patients were divided into groups and subgroups according to the type of they treatment received:

Surgery: this group included patients who received surgery with curative intent, regardless of the neoadjuvant and/or adjuvant treatments administered. Patients receiving different treatments for tumor recurrence were also classified into this group.

Unresectable therapies: this group comprised patients treated pharmacologically for unresectable disease.

Palliative or supportive care: patients in this group were those who did not receive any of the above interventions.

Surgery with curative intent included surgical procedures such as pancreatectomy, pancreatoduodenectomy, excision, and resection of the pancreas (see ICD-9 and ICD-10 codes [35] in Supplementary material Table S2).

Systemic drug treatments included both chemotherapy and biomarker-targeted therapies (VEGF, mTOR) and included any oncological drug registered with the Datamart Billing Service during the study period. Pharmacological treatments were classified as follows.

Neoadjuvant treatment: pharmacological therapies given before surgery with curative intent.

Adjuvant treatment: pharmacological therapies commencing in the first 16 weeks following surgery with curative intent [18].

Treatment for tumor recurrence: pharmacological therapies commencing after the first 16 weeks following surgery with curative intent; following a different therapeutic regimen of adjuvant treatment; or another therapeutic regimen within 12 weeks of completing any adjuvant treatment regimen [18]. Any changes in treatment involving a different active ingredient of the pharmacological therapy were considered another line of treatment (second, third, fourth, and so on).

Treatment for unresectable tumors: these refer to pharmacological therapies in patients not receiving surgery with curative intent. Any changes in treatment involving a different active ingredient of the pharmacological therapy were considered another line of treatment (second, third, fourth, and so on).

Patients’ status at the last follow-up was defined as death or “censored” according to each treatment pattern. Patients lost to follow-up or who had no record of receiving the procedures studied were censored. Survival was defined as the interval from diagnosis (first admission for causes related to pancreatic cancer, first surgery with curative intent for pancreatic cancer, or first systemic antitumor treatment) until death, as notified in the Central Registry of Insured Persons (last follow-up December 2021); this variable was assessed according to age group. Cause of death was not considered because this information is unavailable due to data protection laws.

Finally, we calculated the mean cost per patient and total health expenditure for the first year of treatment and the complete treatment, by treatment pattern, and according to age group. The reimbursement system for the hospital comprises the reimbursement of general hospital care and the economic compensation for highly complex hospital care. To assess surgery costs, we considered the unit price for a curative surgical intervention, as detailed in the Official Gazette of the Government of Catalonia. For procedures performed between January 2014 and July 2017, the unit price in ORDER SLT/79/2014 was used, and between August 2017 and December 2018, the price listed in ORDER SLT/150/2017 was used. The rest of the surgical or therapeutic procedures in inpatients were included in the reimbursement fee for patient discharge, which is adjusted according to hospital complexity. For oncological drug costs, we considered the expenditure attributed to each patient through their anonymous identifier, which uses the reimbursement price of the national health system. Hospital outpatient drugs, which include oncological drugs, are full paid by the Catalan Health Service.

### 2.4. Statistical Analysis

For the descriptive analysis, categorical variables were expressed as absolute and relative frequencies, and continuous variables as means with their standard deviation (SD). Kaplan–Meier curves were used to evaluate survival, with results expressed as medians and 95% confidence interval (CI). Categorical variables were compared using the chi-squared test, and quantitative variables using analysis of variance (ANOVA). The log-rank test was used to compare survival by age group. All statistical analyses were undertaken with SPSS software (v18). A Sankey diagram, developed using the RStudio v4.2.1 tool, was used to analyze treatment patterns.

### 2.5. Ethics

The Committee on Ethics in Animal and Human Experimentation (CEEAH) of the Autonomous University of Barcelona approved the study (CEEAH 4720).

## 3. Results

A total of 4975 patients were diagnosed with a pancreatic malignancy from 2014 to 2018 (Supplementary material Figure S1): 46% (n = 2298) were women, and 55% (n = 2726) were over 70 years old at the time of diagnosis. The most frequent location of the tumor was in the head of the pancreas. Table 1 shows the patients’ characteristics according to age group.

**Figure 1:**
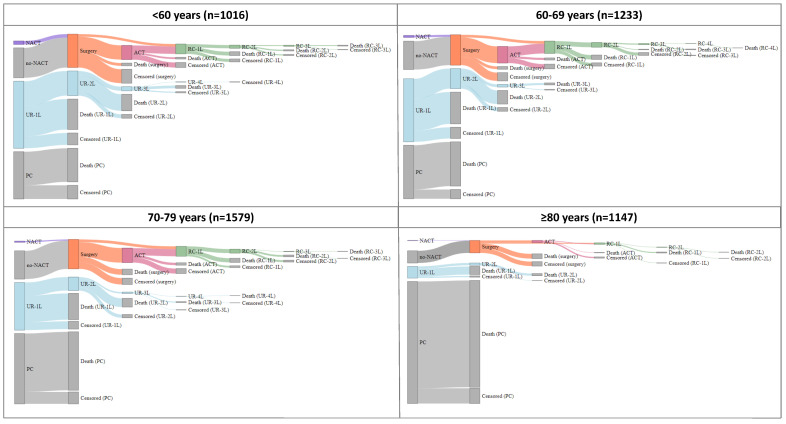
Sankey diagram of the treatment patterns for pancreatic cancer, by age group. NACT: neoadjuvant chemotherapy; ACT: adjuvant chemotherapy; RC–1L: recurrence first-line therapy; RC–2L: recurrence second-line therapy; RC–3L: recurrence third-line therapy; RC–4L: recurrence fourth-line therapy; UR–1L: unresectable first-line therapy; UR–2L unresectable second-line therapy; UR–3L unresectable third-line therapy; UR–4L unresectable fourth-line therapy; UR–5L unresectable fifth-line therapy; PC: palliative or supportive care. Note: The frequencies of patients receiving each treatment by age group are shown in Supplementary material Tables S4–S7. Death (surgery): patient died after surgery; censored (surgery): lost to follow-up or study end after surgery; death (ACT): patient died after ACT; censored (ACT): lost to follow-up or study end after ACT; death (RC–1L, RC–2L, RC–3L or RC–4L): patient died after RC–1L, RC–2L, RC–3L or RC–4L: censored (RC–1L, RC–2L, RC–3L or RC–4L): lost to follow-up or study end after RC–1L, RC–2L, RC–3L or RC–4L; death (UR–1L, UR–2L, UR–3L, UR–4L or UR–5L): patient died after UR–2L, UR–3L, UR–4L or UR–5L: censored (UR–1L, UR–2L, UR–3L, UR–4L or UR–5L): lost to follow-up or study end after UR–1L, UR–2L, UR–3L, UR–4L or UR–5L.

Table 2 and Supplementary material Table S3 describe the groups and subgroups of treatment patterns by age group. Altogether, 18% of the patients underwent surgery with curative intent, 32% received pharmacological treatments for unresectable disease, and 50% received palliative or supportive care. The percentage of patients undergoing surgery with curative intent decreased with age (23% of patients <60 years versus 9% of patients ≥80 years), as did the administration of pharmacological treatment for unresectable disease (45% of patients <60 years versus 8% of patients ≥80 years). In contrast, the proportion of patients with palliative or supportive care increased with age (32% of patients <60 years versus 83% of patients ≥80 years). These differences were statistically significant. In Figure 1, the Sankey diagrams represent the treatment sequences during patient follow-up, according to age group. Likewise, Supplementary material Tables S4–S7 present the frequencies of patients receiving each procedure and the line of treatment according to age group.

**Figure 2:**
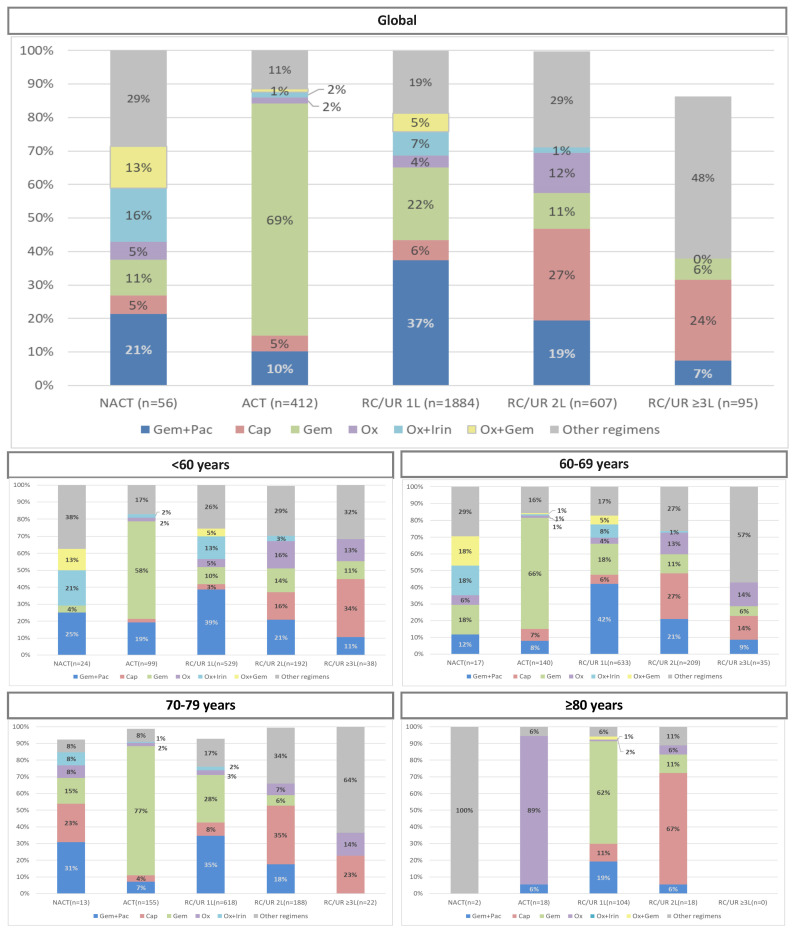
Pharmacological treatments received based on the therapeutic objective and age group. NACT: neoadjuvant chemotherapy; ACT: adjuvant chemotherapy; RC–1L: recurrence first-line therapy; RC–2L: recurrence second-line therapy; RC: recurrence; UR–1L: unresectable first-line therapy, UR–2L: unresectable second-line therapy… Gem: gemcitabine; Pac: paclitaxel; Cap: capecitabine; Ox: Oxaliplatin; Irin: irinotecan. Note: See Supplementary material Tables S8–S12 for other pharmacological treatments with <10% or >100 patients from the total utilization.

Figure 2 shows the distribution of pharmacological treatments used, according to therapeutic objective and age group. Gemcitabine monotherapy was the most widely used adjuvant treatment, and gemcitabine plus paclitaxel was the most frequent regimen for first-line treatment of tumor recurrence or unresectable disease, followed by gemcitabine monotherapy, whose use increased with age; Supplementary material Tables S8–S12 present pharmacological treatments with <10% or >100 patients of the total utilization by age group.

**Figure 3:**
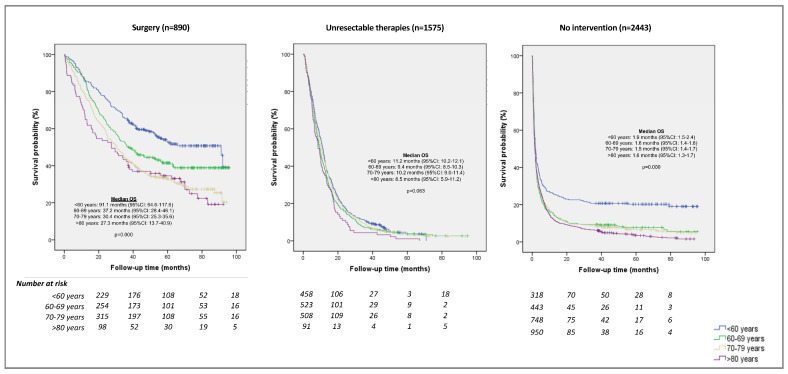
Kaplan–Meier curve for overall survival according to treatment pattern and age. Surgery: patients undergoing surgery with curative intent, regardless of neoadjuvant and/or adjuvant pharmacological treatment; plus patients treated for tumor recurrence. Unresectable therapies: patients receiving pharmacological treatment alone with palliative intent. Palliative or supportive care: patients not receiving any of the treatments described above.

Figure 3 compares overall survival by age group and treatment pattern. For all strategies, median survival decreased with age. Statistically significant differences were observed in the strategies that included surgery, and in those consisting of palliative or supportive care.

Table 3 presents the estimated mean (SD) cost per patient along with the overall health expenditure for year one of treatment and the whole treatment period, according to treatment pattern and age group. In patients treated surgically, the mean cost in the first year decreased with age, from EUR 17,730 (SD 5754) in those under 60 years of age to EUR 15,339 (SD 2634) in patients aged 80 years or older. This decrease was also apparent in patients treated for unresectable disease: EUR 5398 (SD 9581) in the youngest age group versus EUR 1845 (SD 3413) in the oldest. The differences between the means were statistically significant. Likewise, patients who received surgery incurred a total health expenditure of EUR 15.1 million during the first year of treatment compared to EUR 6.8 million in patients with unresectable disease.

## 4. Discussion

In our study, real-world data and clinical practice outcomes were analyzed to provide a comprehensive picture of pancreatic cancer treatments and to illustrate the influence of patient age on treatment patterns, survival, and costs. This design differs from other published studies, which tend to report only on the effects of certain interventions.

In line with other studies [3,36,37], our results show that pancreatic cancer is most frequently diagnosed in elderly patients. However, the older the person is, the less likely they are to receive surgery or pharmacological treatments. The frequent comorbidities, lower functional reserve, and pharmacodynamic changes typical of advanced age usually condition their eligibility for such interventions and raise the risk of adverse reactions, which can limit the dose administered or lead to treatment abandonment [10,38].

The main therapeutic objective in pancreatic cancer is to follow a curative approach, with complete resection (R0) of the primary tumor; the ability to do so depends on its location and relationship with the bile duct and vascular structures [2,5,18]. However, in our cohort, only 18% of patients underwent surgery with curative intent, and this proportion dropped precipitously at more advanced ages. These results, similar to those observed in other studies [3,9,10], show that currently available treatments are insufficient and that there are few therapeutically effective options available for most patients with pancreatic cancer. There is thus a high unmet medical need, both in the early diagnosis of the tumor and in its treatment once diagnosed [15].

Radical surgery is the main therapeutic objective and is potentially curative, so with regard to early detection and diagnosis, further research is necessary to identify biomarkers for the early detection of pancreatic cancer and pre-malignant pancreatic lesions to determine which diagnostic tests are more specific and/or sensitive, and to define the risk groups that should be closely monitored [19,39]. In this line, different European initiatives have been launched, including the use of imaging techniques such as endoscopic ultrasound, magnetic resonance imaging or magnetic resonance cholangiopancreatography [15,40] in individuals with a family history (follow-up from 50–55 years old or 10 years before the youngest relative was diagnosed), or in people with *CDKN2A* or *STK11* mutations, regardless of family history [40]. Likewise, a recent study identified sAXL as a highly specific and sensitive blood biomarker, which allows early detection and discrimination between pancreatic ductal adenocarcinoma and chronic pancreatitis [41]. For this to happen, strategic changes are needed both in the health system and in the Catalan Cancer Plan. Analyzing a more recent time period could contribute to assessing new health system policies implemented to that end.

Regarding pharmacological treatment, European and regional clinical practice guidelines have established the adjuvant treatment regimens to follow, with few changes in the last decade; the recommendations include different mono- or combination therapy schemes according to the functional status of the patient [2,18]. In our study, around 50% of the patients who received surgery were later treated with adjuvant chemotherapy, although in the case of patients over 80 years of age, it was just 18%. The most frequently used pharmacological treatment in patients of any age was gemcitabine monotherapy, which was usually administered to patients with a worse functional status [2,8,18]. In contrast, regimens of FOLFIRINOX or 5-FU, recommended in patients with better functional status [2,8,18], were used infrequently. Older patients were less likely to be candidates for drug treatment for unresectable disease (32% to 45% of patients <80 years vs. 8% of those ≥80 years), which is consistent with previous studies [10].

The most widely used drug treatment in patients under 80 years of age with tumor recurrence or unresectable disease at diagnosis was combination gemcitabine plus paclitaxel, followed by gemcitabine monotherapy, whose use increased with patient age. Gemcitabine monotherapy is the standard of care for locally advanced disease, and combination (nab)-paclitaxel plus gemcitabine or FOLFIRINOX for locally advanced or metastatic disease [2,18]. In general, FOLFIRINOX regimens were uncommon, which probably reflects its high toxicity [2,42,43,44] as well as the age of the patients in our cohort, which was likely associated with a worse functional status and/or comorbidities that contraindicate its use. Another observational study in Sweden reported similar results: the most widely used regimens for patients not candidates for surgery were gemcitabine monotherapy or gemcitabine plus paclitaxel, and a low proportion of patients received FOLFIRINOX [27].

In our study, chemotherapy generally showed modest effectiveness in unresectable disease, with survival of less than one year, which is consistent with the literature and reflects the need for new, more effective pharmacological approaches [8,21,36,45,46,47,48]. Although there are multiple drugs in development that target the stroma, different signaling pathways, and tumor microenvironment, to date, no treatment has provided a clear additional benefit for these patients [5,8,45,48]. Pancreatic cancer usually presents with abundant fibrotic stroma, involving extensive desmoplasia in the primary tumor; both factors are associated with a poor prognosis because they favor the progression of the disease, and limit the access of treatments to the tumor [19,48,49,50]. This fact could explain the resistance to chemotherapy and the failure of targeted vascular endothelial growth factor inhibitor therapy or multikinase inhibitors with antiangiogenic activity [19,48,49,50]. In addition to the presence of desmoplasia, pancreatic cancer also presents an immunosuppressive microenvironment, low T cell infiltration, and low mutational load, factors associated with immunotherapy failure (immune checkpoint inhibitors and cell therapeutic vaccines) [5,8,45,49,50].

Regarding survival, patients who underwent surgery clearly had a better prognosis than those treated pharmacologically for unresectable disease or those receiving palliative or supportive care, as described in the literature [3,9,46,48]. Likewise, in the surgery group, younger patients (<60 years) presented significantly better survival. An international study in the USA and Europe also showed better results in younger patients, with 3-year survival rates of 23% to 39% in those under 60 years old, 16% to 31% in those aged 60–69 years, and 17% to 30% in people 70 years or older [37]. In older patients with unresectable disease amenable to pharmacological treatment, survival was similar to that seen in younger patients, despite age-related pharmacodynamic changes that could favor greater resistance to chemotherapy [10]. This result suggests that age per se is not the determining factor for a poorer prognosis, but rather the associated frailty and disease burden. Other population studies report similar conclusions, observing no differences in patient survival based on age alone [36,46]. Therefore, in this disease context, it is essential to carry out a good oncogeriatric assessment [18] to ensure that treatments are offered to eligible patients according to their functional status, and to avoid making decisions based exclusively on age.

In general, mean treatment costs per patient decrease with age, both in the first year of treatment and overall; this finding is related to the more conservative approaches used in older patients. In Catalonia, surgery costs were calculated based on the unit prices for highly complex procedures delivered in hospital and specialized care settings. These costs rose 4% over the study period. The cost pattern is explained by a greater use of monotherapy treatments in older patients, as well as early discontinuation of these treatments, probably due to their toxicity and the incidence of unmanageable adverse effects in more complex patients [2,10,18,38]. One literature review found that the average cost per patient was higher in other countries than in our study [22]. However, comparing results between these studies is problematic due to differences in the study aims, unit, and reimbursed prices for the drugs. In that line, we agree with Andrade et al. [51] on the need for developing a consensus around the methods applied for estimating these costs.

This study has some limitations. First, some relevant clinical variables were not available in the registries we drew our data from, for example, different tumor-related variables (histology, staging, biomarkers), clinical characteristics (functional status, comorbidities), and treatment data (indication for chemotherapy or palliative radiotherapy). Patients were categorized based on the procedures received, following a pragmatic approach to provide a useful description of clinical practice. The absence of information on patients’ functional status and comorbidities precludes any analysis of how these parameters are related to age or outcomes, also impeding any specific statistical adjustments in that regard. Consequently, the results referring to age groups should be considered in light of this pragmatic approach, not as a causal relationship. A further limitation has to do with the study population, as the sample was selected based on admissions to acute care centers providing care for pancreatic cancer. Furthermore, the clinical terms of the ICD-9 and ICD-10 were modified in the centralized registries of Catalonia during the study period, which may result in misclassification errors, although the classification of patients has been exhaustively reviewed to avoid these. In addition, given the absence of registries that systematically record interventions related to palliative treatment or their costs in long-term health care centers, in our study, we defined only the patterns and determined the costs of pancreatic surgery with curative intent and oncological pharmacological treatments performed in hospitals, as well as the rate paid by the Catalan Health Service to acute hospitals for inpatient and outpatient treatment.

Finally, our healthcare records do not contain data on the participation of patients in clinical trials or on the use of resources in private centers; so, some of the patients in our cohort may have received experimental treatments or undergone surgery or treatment in the private sector. If this information were available, the description of treatment patterns, survival, and related costs would be more accurate. However, in Catalonia, the estimated proportion of oncological surgical interventions in private centers does not exceed 10% of the total, and most pharmacological treatments are administered in the public system, since, unlike the private one, they do not carry any out-of-pocket costs for patients.

## 5. Conclusions

The present study used clinical practice data to provide a broad description of pancreatic cancer treatments in Catalonia, including the use of different treatment patterns and the survival and costs associated with them. Half of the patients diagnosed with pancreatic cancer did not receive specific treatment. Surgery with curative intent was associated with longer survival, although relatively few patients received this intervention, especially at older ages. This finding reaffirms the need to improve diagnosis in early stages of the disease, while the tumor is still resectable, since this is currently the only potentially curative scenario.

Age was inversely related to the use of certain drugs in candidates of any age for drug treatment with unresectable disease; however, survival was similar, so careful oncogeriatric assessment is advisable to ensure the most appropriate indication for eligibility in older patients. More efforts are required to develop more effective and tolerable pharmacological treatments, which allow treating frail patients with unresectable disease and frequent comorbidities at advanced age.

## Figures and Tables

**Table 1:** Characteristics of patients diagnosed with malignant neoplasm of the pancreas.

	Total (N = 4975)		<60 Years (N = 1016)		60–69 Years (N = 1233)		70–79 Years (N = 1579)		≥80 Years (N = 1147)		
	*n*	%	*n*	%	*n*	%	*n*	%	*n*	%	*p* Value *
**Sex**											<0.001
Male	2677	54%	625	62%	764	62%	878	56%	410	36%	
Female	2298	46%	391	38%	469	38%	701	44%	737	64%	
**Tumor site according to ICD-10 codes C25**											
Head of pancreas	2287	46%	419	41%	563	46%	726	46%	579	46%	<0.001
Body of pancreas	467	9%	103	10%	121	10%	159	10%	84	10%	
Tail of pancreas	552	11%	143	14%	156	13%	161	10%	92	10%	
Pancreatic duct	14	0%	2	0%	5	0%	3	0%	4	0%	
Endocrine pancreas	33	1%	13	1%	7	1%	11	1%	2	1%	
Other parts of pancreas	27	1%	4	0%	4	0%	12	1%	7	1%	
Overlapping sites of pancreas	79	2%	16	2%	27	2%	27	2%	9	2%	
Unspecified part of pancreas	1516	30%	316	31%	350	28%	480	30%	370	30%	
**Year of diagnosis**											0.17
2014	906	18%	169	17%	230	19%	300	19%	207	18%	
2015	987	20%	210	21%	231	19%	302	19%	244	21%	
2016	1015	20%	207	20%	255	21%	295	19%	258	22%	
2017	1017	20%	214	21%	248	20%	350	22%	205	18%	
2018	1050	21%	216	21%	269	22%	332	21%	233	20%	

* *p* value determined by chi-square test. The “total” group was excluded from the significance test.

**Table 2:** Treatment patterns for pancreatic cancer, by age group.

	Total (N = 4975)		<60 Years (N = 1016)		60–69 Years (N = 1233)		70–79 Years (N = 1579)		≥80 Years (N = 1147)		
Treatment Patterns	*n*	%	*n*	%	*n*	%	*n*	%	*n*	%	*p* Value *
Surgery ^†^	899	18%	230	23%	255	21%	316	20%	98	9%	<0.0001
Unresectable tumor ^‡^	1593	32%	461	45%	529	43%	511	32%	92	8%	
Palliative or supportive care ^§^	2483	50%	325	32%	449	36%	752	48%	957	83%	

* *p* value determined by chi-square test. The “total” group was excluded from the significance test. ^†^ Patients treated surgically with curative intent, regardless of neoadjuvant and/or adjuvant treatment, plus patients treated for tumor recurrence. ^‡^ Patients treated pharmacologically for non-resectable disease. ^§^ Patients not receiving any of the treatments described above.

**Table 3:** Cost of treatment for pancreatic cancer in EUR, according to treatment pattern and age group.

	Total (N = 4975)				<60 Years (N = 1016)				60–69 Years (N = 1233)				70–79 Years (N = 1579)				≥80 Years (N = 1147)		
	Mean	SD	Total		Mean	SD	Total		Mean	SD	Total		Mean	SD	Total		Mean	SD	Total
**Cost of 1st year**																			
Surgery *	16,833	3965	15,132,436		17,730	5754	4,077,879		16,876	2477	4,303,361		16,607	3512	5,247,969		15,339	2634	1,503,227
Unresectable tumor ^†^	4275	7857	6,809,555		5398	9581	2,488,343		4425	8599	2,341,049		3543	5337	1,810,384		1845	3413	169,778
**Cost of entire treatment**																			
Surgery *	17,615	5093	15,836,300		18,835	7305	4,331,975		17,558	3967	4,477,176		17,416	4307	5,503,454		15,548	2164	1,523,696
Unresectable tumor ^†^	4463	8261	7,108,976		5565	10,157	2,565,313		4687	9009	2,479,179		3706	5564	1,894,010		1853	3412	170,474

SD: standard deviation. *Note*: ANOVA test yielded *p* < 0.001 for all subgroup comparisons by age group. * Patients undergoing surgery with a curative intent, regardless of neoadjuvant and/or adjuvant pharmacological treatment, plus patients treated for tumor recurrence. ^†^ Patients receiving pharmacological treatments alone with a palliative intent.

## Data Availability

Requests to access the data used during the current study should be directed to Programa d’analítica de dades per a la recerca i la innovació en salut (PADRIS): padris@gencat.cat.

## Supplementary Materials

The following supporting information can be downloaded at https://www.mdpi.com/article/10.3390/ijerph20095673/s1, Figure S1: Patient flow chart; Table S1: International Classification of Diseases, 10th revision for malignant neoplasm of pancreas; Table S2. Codes for surgery with curative intent for pancreatic cancer, included in the International Classification of Diseases, 9th and 10th revision; Table S3: Treatment pattern, by age group; Table S4: Treatments used in patients <60 years old with pancreatic cancer; Table S5: Treatments used in patients aged 60–69 years old with pancreatic cancer; Table S6: Treatments used in patients aged 70–79 years old with pancreatic cancer; Table S7: Treatments used in patients aged 80 years or older with pancreatic cancer; Table S8: Pharmacological treatments with <10% or >100 patients of the total utilization; Table S9: Pharmacological treatments with <10% or >100 patients of the total utilization; patients under 60 years of age; Table S10: Pharmacological treatments with <10%or >100 patients of the total utilization; patients aged 60–69 years; Table S11: Pharmacological treatments with <10%or >100 patients of the total utilization; patients aged 70–79 years; Table S12: Pharmacological treatments with <10%or >100 patients of the total utilization; patients aged 80 years or older.

## References

[1] European Comission ECIS—European Cancer Information System. https://ecis.jrc.ec.europa.eu/explorer.php?$0-0$1-AEE$2-All$4-1,2$3-All$6-0,85$5-2008,2008$7-8$CEstByCancer$X0_8-3$CEstRelativeCanc$X1_8-3$X1_9-AE27$CEstBySexByCancer$X2_8-3$X2_-1-1.

[2] Ducreux M., Cuhna A.S., Caramella C., Hollebecque A., Burtin P., Goéré D., Seufferlein T., Haustermans K., Van Laethem J.L., Conroy T. (2015). Cancer of the pancreas: ESMO Clinical Practice Guidelines for diagnosis, treatment and follow-up. Ann. Oncol..

[3] García-Velasco A., Zacarías-Pons L., Teixidor H., Valeros M., Liñan R., Carmona-Garcia M.C., Puigdemont M., Carbajal W., Guardeño R., Malats N. (2020). Incidence and survival trends of pancreatic cancer in Girona: Impact of the change in patient care in the last 25 years. Int. J. Environ. Res. Public Health.

[4] Allemani C., Matsuda T., Di Carlo V., Harewood R., Matz M., Nikšić M., Bonaventure A., Valkov M., Johnson C.J., Estève J. (2018). Global surveillance of trends in cancer survival 2000–14 (CONCORD-3): Analysis of individual records for 37,513,025 patients diagnosed with one of 18 cancers from 322 population-based registries in 71 countries. Lancet.

[5] Gupta R., Amanam I., Chung V. (2017). Current and future therapies for advanced pancreatic cancer. J. Surg. Oncol..

[6] Mizrahi J.D., Surana R., Valle J.W., Shroff R.T. (2020). Pancreatic cancer. Lancet.

[7] McGuigan A., Kelly P., Turkington R.C., Jones C., Coleman H.G., McCain R.S. (2018). Pancreatic cancer: A review of clinical diagnosis, epidemiology, treatment and outcomes. World J. Gastroenterol..

[8] Park W., Chawla A., O’Reilly E.M. (2021). Pancreatic Cancer: A Review. JAMA.

[9] Bengtsson A., Andersson R., Ansari D. (2020). The actual 5-year survivors of pancreatic ductal adenocarcinoma based on real-world data. Sci. Rep..

[10] Higuera O., Ghanem I., Nasimi R., Prieto I., Koren L., Feliu J. (2016). 2016 Pancreatic Cancer: Global view Management of pancreatic cancer in the elderly. World J. Gastroenterol..

[11] Zhao Z.Y., Liu W. (2020). Pancreatic Cancer: A Review of Risk Factors, Diagnosis, and Treatment. Technol. Cancer Res Treat..

[12] Mohile S.G., Dale W., Somerfield M.R., Schonberg M.A., Boyd C.M., Burhenn P.S., Canin B., Cohen H.J., Holmes H.M., Hopkins J.O. (2018). Practical assessment and management of vulnerabilities in older patients receiving chemotherapy: Asco guideline for geriatric oncology. J. Clin. Oncol..

[13] Sourdet S., Brechemier D., Steinmeyer Z., Gerard S., Balardy L. (2020). Impact of the comprehensive geriatric assessment on treatment decision in geriatric oncology. BMC Cancer.

[14] Fusco D., Ferrini A., Pasqualetti G., Giannotti C., Cesari M., Laudisio A., Ballestrero A., Scabini S., Odetti P.R., Colloca G.F. (2021). Comprehensive geriatric assessment in older adults with cancer: Recommendations by the Italian Society of Geriatrics and Gerontology (SIGG). Eur. J. Clin. Investig..

[15] Prades J., Arnold D., Brunner T., Cardone A., Carrato A., Coll-Ortega C., De Luze S., Garel P., Goossens M.E., Grilli R. (2020). Bratislava Statement: Consensus recommendations for improving pancreatic cancer care. ESMO Open.

[16] Bosetti C., Lucenteforte E., Silverman D.T., Petersen G., Bracci P.M., Ji B.T., Negri E., Li D., Risch H.A., Olson S.H. (2012). Cigarette smoking and pancreatic cancer: An analysis from the International Pancreatic Cancer Case-Control Consortium (PANC4). Ann. Oncol..

[17] Lugo A., Peveri G., Bosetti C., Bagnardi V., Crippa A., Orsini N., Rota M., Gallus S. (2018). Strong excess risk of pancreatic cancer for low frequency and duration of cigarette smoking: A comprehensive review and meta-analysis. Eur. J. Cancer.

[18] ICO-ICSPraxis Group (2018). ICO-ICS PRAXI. Per Eltractament Mèdic i Amb Irradiació de L’adenocarcinoma de Pàncrees.

[19] Wild C.P., Weiderpass E., Stewart B.W., World Health Organization (2020). World Cancer Report. Cancer Research for Cancer Prevention.

[20] Coll-Ortega C., Prades J., Manchón-Walsh P., Borras J.M. (2022). Centralisation of surgery for complex cancer diseases: A scoping review of the evidence base on pancreatic cancer. J. Cancer Policy.

[21] Kordes M., Yu J., Malgerud O., Gustafsson Liljefors M., Löhr J.M. (2019). Survival Benefits of Chemotherapy for Patients with Advanced Pancreatic Cancer in A Clinical Real-World Cohort. Cancers.

[22] Carrato A., Falcone A., Ducreux M., Valle J.W., Parnaby A., Djazouli K., Alnwick-Allu K., Hutchings A., Palaska C., Parthenaki I. (2015). A Systematic Review of the Burden of Pancreatic Cancer in Europe: Real-World Impact on Survival, Quality of Life and Costs. J. Gastrointest. Cancer.

[23] Hammers E., Kahn B., Wagner A., Shore C. (2019). Examining the Impact of Real-World Evidence on Medical Product Development.

[24] World Health Organization (2018). Pricing of Cancer Medicines and Its Impacts (WHO).

[25] Garrison L.P., Neumann P.J., Erickson P., Marshall D., Mullins C.D. (2007). Using real-world data for coverage and payment decisions: The ISPOR real-world data Task Force report. Value Health.

[26] Banerjee R., Prasad V. (2020). Are Observational, Real-World Studies Suitable to Make Cancer Treatment Recommendations?. JAMA Netw. Open.

[27] Gränsmark E., Bågenholm Bylin N., Blomstrand H., Fredrikson M., Åvall-Lundqvist E., Elander N.O. (2020). Real World Evidence on Second-Line Palliative Chemotherapy in Advanced Pancreatic Cancer. Front. Oncol..

[28] Koeller J., Surinach A., Arikian Steven R., Zivkovic Marko J.P., Cockrum P., Kim G. (2020). Comparing real-world evidence among patients with metastatic pancreatic ductal adenocarcinoma treated with liposomal irinotecan. Ther. Adv. Med. Oncol..

[29] Sasaki T., Kanata R., Yamada I., Matsuyama M., Ozaka M., Sasahira N. (2019). Improvement of treatment outcomes for metastatic pancreatic cancer: A Real-world Data Analysis. In Vivo.

[30] von Elm E., Altman D.G., Egger M., Pocock S.J., Gøtzsche P.C., Vandenbroucke J.P. (2008). The Strengthening the Reporting of Observational Studies in Epidemiology (STROBE) statement: Guidelines for reporting observational studies. J. Clin. Epidemiol..

[31] World Health Organization (2018). Medicines Reimbursement Policies in Europe.

[32] Generalitat de Catalunya Departament de Salut. Registre Central de població del CatSalut. https://catsalut.gencat.cat/ca/proveidors-professionals/registres-catalegs/registres/central-poblacio/.

[33] Generalitat de Catalunya Departament de Salut. Registres i Catàlegs. https://catsalut.gencat.cat/ca/proveidors-professionals/registres-catalegs/.

[34] Generalitat de Catalunya Departament de Salut. Tecnologies de la Informació i Portal. https://catsalut.gencat.cat/ca/proveidors-professionals/portal-aplicacions/.

[35] Departament de Salut (2018). Generalitat de Catalunya. CIM-10-MC/SCP. Classificació Internacional de Malalties. 10a Revisió. Modificació Clínica. http://www.who.int/classifications/icd/en/.

[36] Brada L.J.H., Walma M.S., van Dam R.M., de Vos-Geelen J., de Hingh I.H., Creemers G.J., Liem M.S., Mekenkamp L.J., de Meijer V.E., de Groot J.W.B. (2021). The treatment and survival of elderly patients with locally advanced pancreatic cancer: A post-hoc analysis of a multicenter registry. Pancreatology.

[37] Huang L., Jansen L., Balavarca Y., Babaei M., van der Geest L., Lemmens V., Van Eycken L., De Schutter H., Johannesen T.B., Primic-Žakelj M. (2018). Stratified survival of resected and overall pancreatic cancer patients in Europe and the USA in the early twenty-first century: A large, international population-based study. BMC Med..

[38] Haanen J.B.A.G., Califano R., Lugowska I., Chiara M., Esmo G., Series H. (2018). Esmo Handbook of Immuno-Oncology.

[39] Khalaf N., El-Serag H.B., Abrams H.R., Thrift A.P. (2021). Burden of Pancreatic Cancer: From Epidemiology to Practice. Clin. Gastroenterol. Hepatol..

[40] Goggins M., Overbeek K.A., Brand R., Syngal S., Del Chiaro M., Bartsch D.K., Bassi C., Carrato A., Farrell J., Fishman E.K. (2020). Management of patients with increased risk for familial pancreatic cancer: Updated recommendations from the International Cancer of the Pancreas Screening (CAPS) Consortium. Gut.

[41] Martínez-Bosch N., Cristóbal H., Iglesias M., Gironella M., Barranco L., Visa L., Calafato D., Jiménez-Parrado S., Earl J., Carrato A. (2022). Soluble AXL is a novel blood marker for early detection of pancreatic ductal adenocarcinoma and differential diagnosis from chronic pancreatitis. eBioMedicine.

[42] Zhang B., Zhou F., Hong J., Ng D.M., Yang T., Zhou X., Jin J., Zhou F., Chen P., Xu Y. (2021). The role of FOLFIRINOX in metastatic pancreatic cancer: A meta-analysis. World J. Surg. Oncol..

[43] Conroy T., Hammel P., Hebbar M., Ben Abdelghani M., Wei A.C., Raoul J.-L., Choné L., Francois E., Artru P., Biagi J.J. (2018). FOLFIRINOX or Gemcitabine as Adjuvant Therapy for Pancreatic Cancer. N. Engl. J. Med..

[44] Chan K.K.W., Guo H., Cheng S., Beca J.M., Redmond-Misner R., Isaranuwatchai W., Qiao L., Earle C., Berry S.R., Biagi J.J. (2020). Real-world outcomes of FOLFIRINOX vs. gemcitabine and nab-paclitaxel in advanced pancreatic cancer: A population-based propensity score-weighted analysis. Cancer Med..

[45] Morrison A.H., Byrne K.T., Vonderheide R.H. (2018). Immunotherapy and Prevention of Pancreatic Cancer. Trends Cancer.

[46] Jung H.A., Han B.R., Kim H.Y., Kim H.J., Zang D.Y., Jung J.Y. (2021). Treatment and Outcomes of Metastatic Pancreatic Cancer in Elderly Patients. Chemotherapy.

[47] Iyikesici M.S. (2020). Long-Term Survival Outcomes of Metabolically Supported Chemotherapy with Gemcitabine-Based or FOLFIRINOX Regimen Combined with Ketogenic Diet, Hyperthermia, and Hyperbaric Oxygen Therapy in Metastatic Pancreatic Cancer. Complement Med. Res..

[48] Neoptolemos J.P., Kleeff J., Michl P., Costello E., Greenhalf W., Palmer D.H. (2018). Therapeutic developments in pancreatic cancer: Current and future perspectives. Nat. Rev. Gastroenterol. Hepatol..

[49] Ren B., Cui M., Yang G., Wang H., Feng M., You L., Zhao Y. (2018). Tumor microenvironment participates in metastasis of pancreatic cancer. Mol. Cancer.

[50] Das S., Shapiro B., Vucic E.A., Vogt S., Bar-Sagi D. (2020). Tumor Cell-Derived IL1β Promotes Desmoplasia and Immune Suppression in Pancreatic Cancer. Cancer Res..

[51] Andrade P., Sacristan J.A., Dilla T. (2017). Health Economics & Outcome Research: The Economic Burden of Cancer in Spain: A Literature Review. Health Econ. Outcome Res. Open Access.

