# Supplementary material for: Management, Survival, and Costs of Pancreatic Cancer: Population-Based Observational Study in Catalonia
Source: Int J Environ Res Public Health. 2023 Apr 28;20(9):5673. doi: 10.3390/ijerph20095673 (PMC10177886; doi:10.3390/ijerph20095673)
Supplement: Supplementary file 1 [file ijerph-20-05673-s001.zip › ijerph-2298747-supplementary.pdf]

Supplementary materials

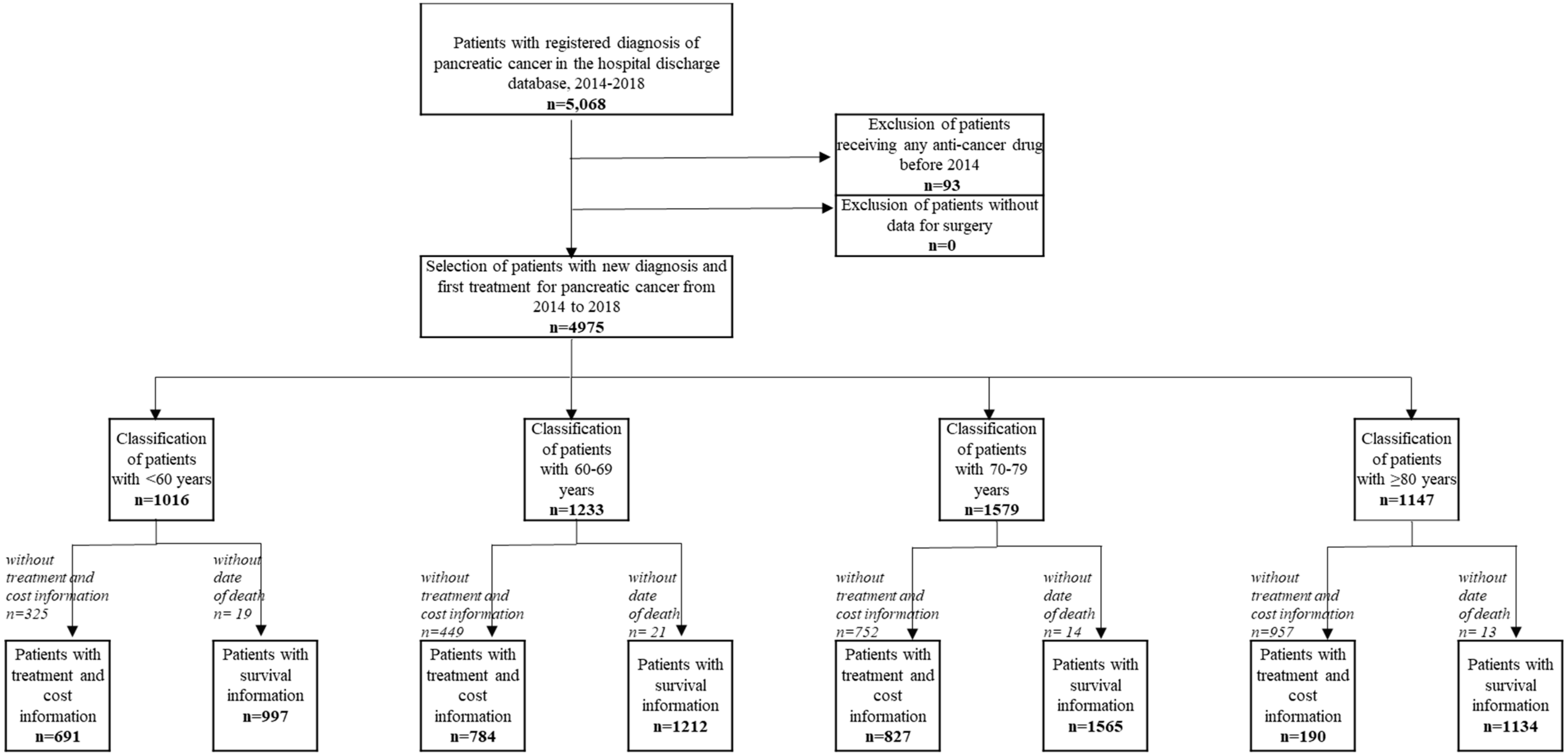

Figure S1. Patient flow chart

Table S1. International Classification of Diseases, 10th revision for malignant neoplasm of pancreas.

Code	
C25	Malignant neoplasm of pancreas
C25.0	Head of pancreas
C25.1	Body of pancreas
C25.2	Tail of pancreas
C25.3	Pancreatic duct
C25.4	Endocrine pancreas (Islets of Langerhans)
C25.7	Other parts of pancreas (Neck of pancreas)
C25.8	Overlapping lesion of pancreas*
C25.9	Pancreas, unspecified

Note: Adapted from https://icd.who.int/browse10/2019/en#/; *A neoplasm that overlaps two or more contiguous sites within a three-character category and whose point of origin cannot be determined.

Table S2. Codes for surgery with curative intent for pancreatic cancer, included in the International Classification of Diseases, 9th and 10th revision.

ICD-9 code	Description
52.21	Endoscopic excision or destruction of lesion or tissue of pancreatic duct
52.22	Other excision or destruction of lesion or tissue of pancreas or pancreatic duct
52.51	Proximal pancreatectomy
52.52	Distal pancreatectomy
52.53	Radical subtotal pancreatectomy
52.59	Other partial pancreatectomy
52.6	Total pancreatectomy
52.7	Radical pancreaticoduodenectomy
**ICD-10 codes**	**Description**
0F5D0ZZ	Destruction of pancreatic duct, open approach
0F5G0ZZ	Destruction of pancreas, open approach
0F5G3ZZ	Destruction of pancreas, percutaneous approach
0FBG0ZZ	Excision of pancreas, open approach
0FBG3ZZ	Excision of pancreas, percutaneous approach
0FBG4ZZ	Excision of pancreas, percutaneous endoscopic approach
0FTG0ZZ	Resection of pancreas, open approach
0FTG4ZZ	Resection of pancreas, percutaneous endoscopic approach

Note: Adapted from https://eciemaps.mscbs.gob.es/ecieMaps/browser/index_9_mc.html https://eciemaps.mscbs.gob.es/ecieMaps/browser/index_10_pcs.html.

Table S3. Treatment pattern, by age group.

Age (years)	Treatment pattern	n	%
**Global (n=4975)**	Surgery*	453	9%
	NACT	34	1%
	NACT-ACT	22	0%
	ACT	390	8%
	Unresectable tumor†	1593	32%
	Palliative or supportive care‡	2483	50%
**<60 years (n=1016)**	Surgery*	116	11%
	NACT	16	9%
	NACT-ACT	8	2%
	ACT	90	1%
	Unresectable tumor†	461	45%

	Palliative or supportive care‡	325	32%
**60-69 years (n=1233)**	Surgery*	106	9%
	NACT	9	1%
	NACT-ACT	8	1%
	ACT	132	11%
	Unresectable tumor†	529	43%
	Palliative or supportive care‡	449	36%
**70-79 years (n=1579)**	Surgery*	153	10%
	NACT	7	0%
	NACT-ACT	6	0%
	ACT	150	9%
	Unresectable tumor†	511	32%
	Palliative or supportive care‡	752	48%
**≥80 years (n=1147)**	Surgery*	78	7%
	NACT	2	0%
	NACT-ACT	0	0%
	ACT	18	2%
	Unresectable tumor†	92	8%
	Palliative or supportive care‡	957	83%

NACT: neoadjuvant chemotherapy; administered prior to surgery; can also include different lines of treatment for tumor recurrence. NACT-ACT: neoadjuvant and adjuvant chemotherapy; administered prior to surgery; can also include different lines of treatment for tumor recurrence. ACT: adjuvant chemotherapy; administered prior to surgery; can also include different lines of treatment for tumor recurrence. *Patients receiving surgery with a curative intent; can also include different lines of treatment for tumor recurrence; †Patients receiving pharmacological treatments for an unresectable tumor. § Patients who do not receive any of the treatments described above.

Table S4. Treatments used in patients <60 years old with pancreatic cancer.

**Treatments**		**Patients (n)**	**Patients (%)**
NACT	Surgery	24	10%
No NACT	Surgery	206	90%
Surgery	ACT	98	43%
	RC-1L	17	7%
	Death	19	8%
	Censored	96	42%
ACT	RC-1L	51	52%
	Death	12	12%
	Censored	35	36%
RC-1L	RC-2L	22	32%
	Death	26	38%
	Censored	20	29%
RC-2L	RC-3L	6	27%
	Death	8	36%
	Censored	8	36%
RC-3L	RC-4L	0	0%
	Death	4	67%
	Censored	2	33%
RC-4L	UR-5L	0	0%
	Death	0	0%
	Censored	0	0%
UR-1L	UR-2L	170	37%

	Death	209	45%
	Censored	82	18%
UR-2L	UR-3L	29	17%
	Death	114	67%
	Censored	27	16%
UR-3L	UR-4L	2	7%
	Death	19	66%
	Censored	8	28%
UR-4L	UR-5L	0	0%
	Death	0	0%
	Censored	2	100%
PC	Death	231	71%
	Censored	94	29%

NACT: neoadjuvant chemotherapy; ACT: adjuvant chemotherapy; RC: recurrence therapy; UR: treatment for unresectable tumor; PC: palliative or supportive care.

Table S5. Treatments used in patients aged 60-69 years old with pancreatic cancer.

Treatments		Patients (n)	Patients (%)
NACT	Surgery	17	7%
No NACT	Surgery	238	93%
Surgery	ACT	140	55%
	RC-1L	22	9%
	Death	23	9%
	Censored	70	27%
ACT	RC-1L	82	59%
	Death	18	13%
	Censored	40	29%
RC-1L	RC-2L	40	38%
	Death	37	36%
	Censored	27	26%
RC-2L	RC-3L	10	25%
	Death	5	13%
	Censored	25	63%
RC-3L	RC-4L	1	10%
	Death	6	60%
	Censored	3	30%
RC-4L	UR-5L	0	0%
	Death	1	100%
	Censored	0	0%
UR-1L	UR-2L	169	32%
	Death	266	50%
	Censored	94	18%
UR-2L	UR-3L	23	14%
	Death	114	67%
	Censored	32	19%
UR-3L	UR-4L	0	0%
	Death	17	74%
	Censored	6	26%
UR-4L	UR-5L	0	0%
	Death	0	0%
	Censored	0	0%

PC	Death	371	83%
	Censored	78	17%

NACT: neoadjuvant chemotherapy; ACT: adjuvant chemotherapy; RC: recurrence therapy; UR: treatment for unresectable tumor; PC: palliative or supportive care.

Table S6. Treatments used in patients aged 70-79 years old with pancreatic cancer.

Treatments		Patients (n)	Patients (%)
NACT	Surgery	13	4%
No NACT	Surgery	303	96%
Surgery	ACT	156	49%
	RC-1L	30	9%
	Death	61	19%
	Censored	68	22%
ACT	RC-1L	77	49%
	Death	26	17%
	Censored	53	34%
RC-1L	RC-2L	43	40%
	Death	45	42%
	Censored	19	18%
RC-2L	RC-3L	4	9%
	Death	19	44%
	Censored	20	47%
RC-3L	RC-4L	0	0%
	Death	2	50%
	Censored	2	50%
UR-1L	UR-2L	145	28%
	Death	282	55%
	Censored	84	16%
UR-2L	UR-3L	16	11%
	Death	94	65%
	Censored	35	24%
UR-3L	UR-4L	2	13%
	Death	11	69%
	Censored	3	19%
UR-4L	UR-5L	0	0%
	Death	1	50%
	Censored	1	50%
PC	Death	624	83%
	Censored	128	17%

NACT: neoadjuvant chemotherapy; ACT: adjuvant chemotherapy; RC: recurrence therapy; UR: treatment for unresectable tumor; PC: palliative or supportive care.

Table S7. Treatments used in patients aged 80 years or older with pancreatic cancer.

Treatments		Patients (n)	Patients (%)
NACT	Surgery	2	2%
No NACT	Surgery	96	98%
Surgery	ACT	18	18%
	RC-1L	7	7%
	Death	38	39%
	Censored	35	36%
ACT	RC-1L	5	28%

	Death	3	17%
	Censored	10	56%
RC-1L	RC-2L	2	17%
	Death	8	67%
	Censored	2	17%
RC-2L	RC-3L	0	0%
	Death	1	50%
	Censored	1	50%
UR-1L	UR-2L	18	20%
	Death	64	70%
	Censored	10	11%
UR-2L	UR-3L	0	0%
	Death	16	89%
	Censored	2	11%
PC	Death	837	87%
	Censored	120	13%

NACT: neoadjuvant chemotherapy; ACT: adjuvant chemotherapy; RC: recurrence therapy; UR: treatment for unresectable tumor; PC: palliative or supportive care.

Table S8. Pharmacological treatments with <10% or >100 patients of the total utilization.

**Neoadjuvant therapies (n=56)**	
Irinotecan/oxaliplatin + capecitabine	7%
Irinotecan/oxaliplatin + gemcitabine	5%
Paclitaxel	4%
Capecitabine + gemcitabine	2%
Capecitabine + paclitaxel	2%
Cisplatin + vinorelbine	2%
Oxaliplatin + gemcitabine + paclitaxel	4%
Oxaliplatin + capecitabine + gemcitabine	2%
Oxaliplatin + 5-FU	2%
**Adjuvant therapies (n=412)**	
Capecitabine + gemcitabine	11%
5-FU	<1%
Cisplatin + vinorelbine	<1%
FOLFIRNOX	<1%
Irinotecan	<1%
Oxaliplatin + capecitabine + gemcitabine + paclitaxel	<1%
Pembrolizumab	<1%
Paclitaxel	<1%
**First-line treatment for recurrence or unresectable tumors (n=1882)**	
FOLFIRNOX	2%
Paclitaxel	2%
Capecitabine + gemcitabine	2%
5-FU/oxaliplatin	2%
Cisplatin + gemcitabine	1%
Cisplatin	1%
Everolimus	1%
Oxaliplatin + capecitabine	1%
5-FU	1%
Irinotecan	1%
Sunitinib	1%

Cisplatin + etoposide	<1%
Etoposide	<1%
Docetaxel	<1%
5-FU/oxaliplatin + gemcitabine	<1%
Irinotecan/oxaliplatin + capecitabine	<1%
Vinorelbine	<1%
5-FU/irinotecan/oxaliplatin	<1%
Carboplatin + etoposide	<1%
Pemetrexed	<1%
Cisplatin + gemcitabine + paclitaxel	<1%
Irinotecan/oxaliplatin + gemcitabine	<1%
5-FU/irinotecan	<1%
5-FU + everolimus	<1%
Capecitabine + paclitaxel	<1%
FOLFIRNOX + gemcitabine	<1%
Irinotecan + gemcitabine	<1%
Oxaliplatin + cisplatin + gemcitabine	<1%
5-FU/oxaliplatin + capecitabine	<1%
5-FU/oxaliplatin + cisplatin	<1%
5-FU/oxaliplatin + paclitaxel	<1%
5-FU + carboplatin	<1%
5-FU + cisplatin	<1%
5-FU + gemcitabine	<1%
Bevacizumab	<1%
Capecitabine + bevacizumab	<1%
Capecitabine + sunitinib	<1%
Carboplatin + /-cisplatin + etoposide	<1%
Carboplatin + paclitaxel	<1%
Cetuximab	<1%
Cisplatin/carboplatin	<1%
Cisplatin + docetaxel	<1%
Cisplatin + paclitaxel	<1%
Cisplatin + vinorelbine	<1%
Dabrafenib	<1%
FOLFIRNOX + capecitabine	<1%
Gemcitabine + erlotinib	<1%
Irinotecan/oxaliplatin + capecitabine + gemcitabine	<1%
Irinotecan + capecitabine	<1%
Irinotecan + gemcitabine + paclitaxel	<1%
Oxaliplatin + capecitabine + bevacizumab	<1%
Paclitaxel + bevacizumab	<1%
Paclitaxel + cetuximab	<1%
Pembrolizumab	<1%
**Second-line treatment for recurrence or unresectable tumors (n=607)**	
Irinotecan	6%
5-FU + oxaliplatin	5%
Paclitaxel	4%
5-FU	3%
Oxaliplatin + capecitabine	3%
5-FU + irinotecan	1%
Everolimus	1%
Sunitinib	1%
FOLFIRNOXç	1%

Nivolumab	1%
Docetaxel	<1%
Capecitabine + gemcitabine	<1%
Cisplatin	<1%
Gemcitabine/paclitaxel	<1%
5-FU + cisplatin	<1%
Atezolizumab	<1%
Bevacizumab	<1%
Bevacizumab + cetuximab	<1%
Capecitabine + paclitaxel	<1%
Carboplatin	<1%
Carboplatin + pemetrexed	<1%
Cetuximab	<1%
Cisplatin + etoposide	<1%
Etoposide	<1%
Irinotecan + capecitabine	<1%
Irinotecan + gemcitabine	<1%
Pembrolizumab	<1%
Topotecan	<1%
**Third- or higher-line treatment for recurrence or unresectable tumors (n=98)**	
Irinotecan (21%)	21%
Paclitaxel	9%
5-FU	6%
5-FU + oxaliplatin	3%
Sunitinib	2%
5-FU + irinotecan	1%
Carboplatin + etoposide	1%
Cetuximab	1%
Cisplatin + gemcitabine	1%
Erlotinib	1%
Oxaliplatin + capecitabine	1%

Table S9. Pharmacological treatments with <10% or >100 patients of the total utilization; patients under 60 years of age.

**Neoadjuvant therapies (n=24)** Irinotecan/oxaliplatin + capecitabine	8%
Irinotecan/oxaliplatin + gemcitabine	8%
Capecitabine + paclitaxel	4%
Cisplatin + vinorelbine	4%
Oxaliplatin + gemcitabine + paclitaxel	4%
Oxaliplatin/5-FU	4%
**Adjuvant therapies (n=99)**	
5-FU	1%
FOLFIRNOX	1%
Paclitaxel	1%
Pembrolizumab	1%
**First-line treatment for recurrence or unresectable tumors (n=528)**	
FOLFIRNOX	4%
5-FU + oxaliplatin	2%
Capecitabine + gemcitabine	2%
Everolimus	2%
Cisplatin	2%

Paclitaxel	2%
5-FU	2%
Cisplatin + etoposide	1%
Cisplatin + gemcitabine	1%
Sunitinib	1%
5-FU + oxaliplatin + gemcitabine	1%
Irinotecan	1%
Irinotecan + oxaliplatin + gemcitabine	1%
Oxaliplatin + capecitabine	1%
5-FU + irinotecan	<1%
5-FU + everolimus	<1%
Carboplatinetoposide	<1%
Oxaliplatin + cisplatin + gemcitabine	<1%
5-FU + irinotecan + oxaliplatin	<1%
Bevacizumab	<1%
Capecitabine + sunitinib	<1%
Carboplatin/cisplatin + etoposide	<1%
Cisplatin + gemcitabine + paclitaxel	<1%
Cisplatin + paclitaxel	<1%
Cisplatin + vinorelbine	<1%
Irinotecan + oxaliplatin + capecitabine + gem-	<1%
citabine	<1%
Pembrolizumab	<1%
Pemetrexed	<1%
Vinorelbine	
**Second-line treatment for recurrence or unresectable tumors (n=192)**	
Irinotecan	6%
5-FU + oxaliplatin	5%
5-FU	4%
Paclitaxel	4%
Oxaliplatin + capecitabine	3%
FOLFIRNOX	2%
Everolimus	1%
5-FU + irinotecan	1%
Atezolizumab	1%
Bevacizumab	1%
Carboplatin	1%
Cisplatin	1%
Cisplatin + etoposide	1%
Docetaxel	1%
Everolimus + sunitinib	1%
Sunitinib	1%
**Third- or higher-line treatment for recurrence or unresectable tumors (n=38)**	
Irinotecan	18%
5-FU	8%
Paclitaxel	3%
Sunitinib	3%

Table S10. Pharmacological treatments with <10%or >100 patients of the total utilization; patients aged 60-69 years.

**Neoadjuvant therapies (n=17)**	
Irinotecan/oxaliplatin + capecitabine	12%

Oxaliplatin + capecitabine + gemcitabine	6%
Paclitaxel	6%
Irinotecan/oxaliplatin + gemcitabine	6%
**Adjuvant therapies (n=140)**	
Capecitabine + gemcitabine	14%
Paclitaxel	1%
Oxaliplatin + capecitabine	1%
**First-line treatment for recurrence or unresectable tumors (n=633)**	
Paclitaxel	2%
Capecitabine + gemcitabine	2%
Cisplatin + gemcitabine	1%
FOLFIRNOX	1%
Oxaliplatin + capecitabine	1%
Irinotecan	1%
Cisplatin	1%
5-FU	1%
5-FU/oxaliplatin	1%
Etoposide	1%
Docetaxel	<1%
Irinotecan/oxaliplatin + capecitabine	<1%
5-FU/oxaliplatin + gemcitabine	<1%
Everolimus	<1%
FOLFIRNOX + gemcitabine	<1%
5-FU/irinotecan/oxaliplatin	<1%
5-FU/oxaliplatin + capecitabine	<1%
5-FU + carboplatin	<1%
5-FU + cisplatin	<1%
Carboplatinetoposide	<1%
Cisplatin/carboplatin	<1%
Cisplatin + docetaxel	<1%
Cisplatin + etoposide	<1%
FOLFIRNOX + capecitabine	<1%
Gemcitabine + erlotinib	<1%
Irinotecan + capecitabine	<1%
Irinotecan + gemcitabine	<1%
Oxaliplatin + capecitabine + bevacizumab	<1%
Paclitaxel + bevacizumab	<1%
Pemetrexed	<1%
Vinorelbine	<1%
**Second-line treatment for recurrence or unresectable tumors (n=209)**	
Irinotecan	6%
5-FU/oxaliplatin	5%
Paclitaxel	4%
Oxaliplatin + capecitabine	4%
5-FU	3%
Nivolumab	2%
Docetaxel	1%
Everolimus	1%
Sunitinib	1%
5-FU/irinotecan	1%
5-FU + cisplatin	1%

Bevacizumab + cetuximab	1%
Capecitabine + gemcitabine	1%
Capecitabine + paclitaxel	1%
Carboplatinpemetrexed	1%
Cetuximab	1%
Etoposide	1%
Irinotecan/oxaliplatin	1%
Irinotecan + capecitabine	1%
Pembrolizumab	1%
Topotecan	1%
**Third- or higher-line treatment for recurrence or unresectable tumors (n=35)**	
Irinotecan	17%
Paclitaxel	9%
5-FU + oxaliplatin	9%
5-FU	6%
Cetuximab	3%
Cisplatin + gemcitabine	3%
Oxaliplatin + capecitabine	3%
Carboplatin + etoposide	3%
Paclitaxel	3%
Sunitinib	3%

Table S11. Pharmacological treatments with <10%or >100 patients of the total utilization; patients aged 70-79 years.

**Neoadjuvant therapies (n=13)**	
Irinotecan + oxaliplatin + capecitabine	8%
**Adjuvant therapies (n=155)**	
Capecitabine + gemcitabine	7%
**First-line treatment for recurrence or unresectable tumors (n=617)**	
5-FU + oxaliplatin	2%
Capecitabine + gemcitabine	1%
Oxaliplatin + capecitabine	1%
Cisplatin + gemcitabine	1%
Paclitaxel	1%
Everolimus	1%
Cisplatin	1%
FOLFIRNOX	1%
Sunitinib	1%
5-FU	1%
Etoposide	<1%
Vinorelbine	<1%
5-FU + irinotecan + oxaliplatin	<1%
Capecitabine + paclitaxel	<1%
Cisplatin + gemcitabine + paclitaxel	<1%
Docetaxel	<1%
Irinotecan	<1%
Irinotecan + oxaliplatin + capecitabine	<1%
Pemetrexed	<1%
5-FU + oxaliplatin + cisplatin	<1%
5-FU + oxaliplatin + paclitaxel	<1%
5-FU + gemcitabine	<1%
Capecitabine + bevacizumab	<1%

Carboplatin + paclitaxel	<1%
Carboplatinetoposide	<1%
Cetuximab	<1%
Cisplatin + etoposide	<1%
Dabrafenib	<1%
Irinotecan + gemcitabine	<1%
Irinotecan + gemcitabine + paclitaxel	<1%
Paclitaxel + cetuximab	<1%
**Second-line treatment for recurrence or unresectable tumors (n=188)**	
Irinotecan	6%
5-FU + oxaliplatin	5%
Paclitaxel	4%
Oxaliplatin + capecitabine	4%
5-FU	3%
Nivolumab	2%
Docetaxel	1%
Everolimus	1%
Sunitinib	1%
5-FU + irinotecan	1%
5-FU + cisplatin	1%
Bevacizumab + cetuximab	1%
Capecitabine + gemcitabine	1%
Capecitabine + paclitaxel	1%
Carboplatin + pemetrexed	1%
Cetuximab	1%
Etoposide	1%
Irinotecan + capecitabine	1%
Pembrolizumab	1%
Topotecan	1%
**Third- or higher-line treatment for recurrence or unresectable tumors (n=22)**	
Irinotecan	32%
Paclitaxel	18%
5-FU	5%
5-FU + irinotecan	5%
Erlotinib	5%

Table S12. Pharmacological treatments with <10%or >100 patients of the total utilization; patients aged 80 years or older.

**Neoadjuvant therapies (n=2)**	
Capecitabine + gemcitabine	50%
Paclitaxel	50%
**Adjuvant therapies (n=18)**	
NA	
**First-line treatment for recurrence or unresectable tumors (n=104)**	
Everolimus	2%
Capecitabine + gemcitabine	1%
Docetaxel	1%
Etoposide	1%
Paclitaxel	1%
**Second-line treatment for recurrence or unresectable tumors (n=18)**	
Irinotecan	6%
Paclitaxel	6%

Third- or higher-line treatment for recurrence or unresectable tumors (n=0)
NA

NA: not applicable.
